# Plasmonic Slippery Surface for Surface-Enhanced Raman Spectroscopy and Protein Adsorption Inhibition

**DOI:** 10.1021/acs.analchem.4c01844

**Published:** 2025-01-28

**Authors:** Swithin Hanosh, Monisha K, Sajan D. George

**Affiliations:** † Department of Atomic and Molecular Physics, Manipal Academy of Higher Education, Manipal 576104, India; ‡ Centre for Applied Nanosciences (CANs), Manipal Academy of Higher Education, Manipal 576104, India

## Abstract

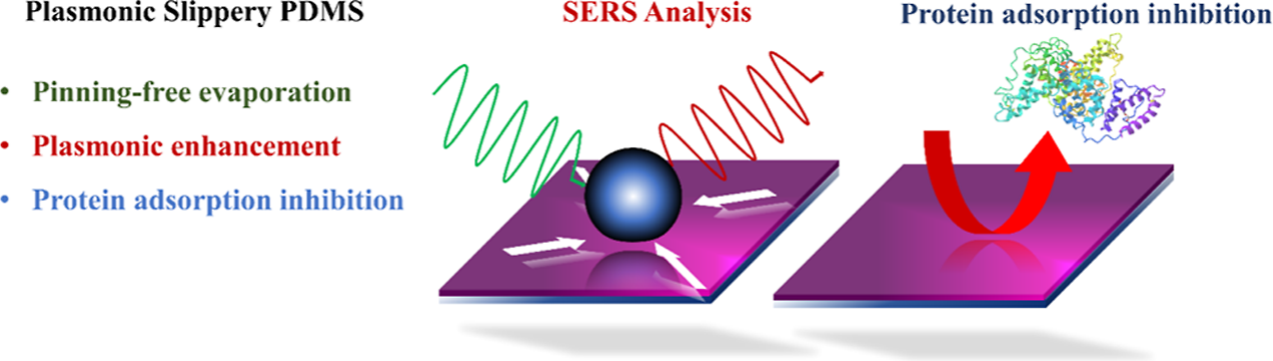

Slippery liquid-infused porous surfaces (SLIPSs) are a class of surface that offers low contact angle hysteresis and low tilt angle for water droplet shedding. This property also endows the surface with pinning-free evaporation, which in turn has been exploited for analyte concentration enrichment for Surface Enhanced Raman Spectroscopic applications and antibiofouling. Herein, we demonstrate a facile approach for creating SLIPS with low contact angle hysteresis and low tilt angle for water shedding by coating the equal-volume mixture of polydimethylsiloxane (PDMS) and silicone oil. By exploiting the in situ plasmonic particle reduction capability of the PDMS, the surface is converted to plasmonic SLIPS, which illustrates its potential as a sensitive analytical platform via surface-enhanced Raman spectroscopy. The Raman spectroscopic studies using crystal violet as a reference sample show a limit of detection of 76 pM. Further, we have demonstrated that the fabricated plasmonic substrate is found to be more efficient in inhibiting proteins (bovine serum albumin) on the surface compared to pristine PDMS surfaces. Our fabricated plasmonic surface can find applications in ultrasensitive molecular detection for applications related to analytical chemistry, diagnostics, environmental monitoring, and national security and more importantly can control the nonspecific adsorption of proteins.

## Introduction

Inspired by naturally occurring surfaces, biomimicking the surface properties of the materials to achieve interfaces with unique properties has been the subject of intense research due to its applications. Driven by the plethora of applications in the field of self-cleaning,^1−3^ anti-icing,^4−6^ antifouling,^7−9^ condensation,^10−12^ and water harvesting,^13−15^ much research has been focused on the fabrication of superhydrophobic and slippery lubricant-infused porous surfaces (SLIPSs). While the majority of the superhydrophobic surfaces rely on the trapped air pocket explained in the Cassie–Baxter state of wetting, the SLIPS is based on the intrusion of the oil layer that replaces the air in the porous network of the substrate.^16,17^ Apart from the aforementioned applications, by coupling with plasmonic nanoparticles, these surfaces are currently employed for spectroscopic and plasmonic photocatalytic applications.^18,19^ The high contact angle and low contact angle hysteresis (CAH) in the case of superhydrophobic surface facilitate the concentration enrichment of the analyte to a small region of the substrate and thereby improving the limit of detection (LOD) substantially.^20^ In addition, the low CAH of lotus-leaf-inspired superhydrophobic surfaces as well as SLIPSs provides a substrate that exhibits no coffee-ring effect following the evaporation due to the nonpinning nature of the substrate.^17^ However, superhydrophobic surfaces with ultralow CAH require hierarchical micronanoscale structures, and the stability of such structures for practical applications is a great concern and often difficult to achieve cost-effectively. On the other hand, liquid infusion in SLIPSs is often associated with difficulties such as depletion of the oil layer during repeated usage and masking of structural features of the substrates by the oil film.^21^ Therefore, of late, nonadhesive polymer layer grafting is being explored to create nonadhesive surfaces for practical applications such as water harvesting, liquid droplet assay, anticorrosive coatings, etc.^13,22,23^ Despite the promise such substrates hold, the high pinning and low droplet mobility often lead to undesired effects and these substrates often exhibit nonadhesive nature above a threshold tilt angle. In addition, the spectroscopic application of such substrates requires the incorporation of plasmonic particles into the substrate. Efforts are now being put to create a facile approach of fabricating surfaces that incorporate the plasmonic particles into the substrates while retaining their slippery behavior so that it can be employed for plasmon-enhanced sensing platforms such as surface-enhanced Raman spectroscopy (SERS). Such surfaces may mitigate the issues of uneven adsorption of analyte molecules in conventional colloidal assembly-based SERS substrates.

The conventional SERS detection of analyte molecules involves the deposition of a droplet solution containing analyte molecules onto the plasmonic substrate and probing of the Raman signal from the molecules following the evaporation of the droplet. However, most of the fabricated SERS substrates are hydrophilic in nature, and the droplet spreads to the surface and results in deposition of analyte molecules throughout the substrate, forming coffee-ring stains. Therefore, the reproducibility of the Raman signal depends on many factors such as droplet shape, surface charge of the substrate, substrate wettability, etc. Moreover, the Raman signal enhancement in the SERS studies depends on the availability of analyte molecules in the hotspot region of the substrate. Although, in the drop coating deposition Raman technique,^24,25^ the coffee-ring phenomenon is exploited for Raman spectroscopic studies, wherein microlitre volume of analyte solution is dispensed and dried on hydrophobic surfaces, the evaporation of the droplets onto a superhydrophobic surface wherein the contact area of the droplet with the substrate is minimum improves the concentration enrichment of analyte molecules into a smaller region compared to a hydrophobic surface. However, the fabrication of such superhydrophobic substrates with hierarchical micro and nanoscale structures normally requires expensive E-beam lithography, optical lithography, laser writing, physical vapor deposition, and reactive ion etching techniques.^20,26−28^ Therefore, the development of alternate cost-effective strategies to create SLIPSs that allow pinning-free evaporation for the concentration enrichment of analyte molecules is of paramount importance in developing cost-effective SERS analytical devices. Protein fouling on surfaces is another widespread issue encountered in biomedical settings, and thus, the phenomenon of protein adsorption on surfaces due to the attraction of molecules of bulk proteins immersed in an aqueous environment has to be considered for bioapplications. Upon adsorption of the protein, the molecules lose their degrees of freedom and undergo biochemical and physiochemical changes, and the adsorption is often irreversible. The adsorption of proteins onto the SLIPS is not an area that has been widely explored.

Herein, we illustrate the fabrication of a plasmonic slippery surface in a simple two-step process. In the first step, poly(dimethylsiloxane) (PDMS) and silicone oil are mixed in an equal volume ratio and thermally cured to obtain slippery PDMS. In the second step, the plasmonic slippery surface is fabricated via the in situ reduction of the plasmonic particles onto the slippery PDMS surface. Following the characterization of the surface properties of the plasmonic slippery surface via X-ray diffraction (XRD) studies and optical spectroscopic techniques, the potential of such surfaces for SERS detection of analyte is illustrated by taking crystal violet as a reference sample. Further, a comparative study of the protein adsorption onto the plain PDMS, slippery PDMS, and the plasmonic slippery surface is also investigated and results unambiguously illustrate the role of the wetting nature of the surface on protein adsorption by taking bovine serum albumin (BSA) as a model protein.

## Materials and Methods

### Chemicals

18.2 MΩ cm ultrapure milli-Q water was used for the preparation of all solutions. All of the chemical reagents mentioned below were used without further purification. The PDMS prepolymer was purchased from Dow Corning. Silicone oil (500 cSt), gold(III) chloride, and albumin–fluorescein isothiocyanate conjugate (FITC-BSA) were obtained from Sigma-Aldrich. Crystal violet was purchased from Loba Chemie Private Limited. BSA and Phosphate Buffer Saline (PBS) were obtained from Himedia. All glassware was cleaned with piranha solution and milli-Q water before use.

### Preparation of Slippery PDMS Coating

One mL portion of PDMS (10:1) was magnetically stirred with 1 mL of silicone oil (500 cSt viscosity) for 10 min to obtain a homogeneous solution. The mixture was degassed to remove any air bubbles trapped within the mixture. A small drop of the mixture was taken and poured over the glass surface and spin-coated at 1500 rpm for 15 s to obtain a uniform coating over the surface. The glass substrate with the coating was cured at 80 °C for 24 h in air to obtain a slippery PDMS coating.

### Preparation of Plasmonic Slippery PDMS Coating

The slippery PDMS-coated glass substrate was immersed in 5 mL of 20 mM gold chloride solution diluted with 1 mL of acetone for 48 h for in situ reduction of gold nanoparticles onto the slippery PDMS. As compared to the slippery PDMS surface, the reduction of gold particles onto the slippery surface manifested a color change to pinkish-red after 48 h.

### Characterization

The crystallinity of the slippery PDMS surface, as well as the formation of the gold nanoparticles onto the slippery PDMS surface, was probed via XRD studies (Model: Ultima IV, Rigaku). Further, the surfaces were characterized using a commercially available UV–vis absorption spectrometer (PerkinElmer Lambda 950), a Fourier transform infrared spectroscopy instrument in the attenuated total reflection mode (ATR-FTIR, FT/IR 6300, JASCO), and a home-built micro-Raman spectroscopy system. The surface wettability of the samples was characterized using a commercially available contact angle goniometer (Holmarc, India, model no.: HO-IAD-CAM-01A). The fluorescence intensity measurements were carried out using an upright microscope (Nikon Eclipse Ni-E).

### Protein Adhesion Test

The prepared substrates were immersed in 1 mg/mL FITC-BSA in PBS solution and incubated at 37 °C for 1 day, 2 days, and 3 days. After the substrates were rinsed with PBS solution to remove weakly adhered proteins, the substrates were observed under an upright microscope. The images were obtained under similar parameters, and the fluorescence intensities were quantified using ImageJ software. At least five images were taken for each sample.

## Results and Discussion

Herein, the effort is made to combine the advantage of an enhanced Raman signal from a plasmonic substrate and the concentration enrichment of the SLIPSs to achieve a better LOD of the analyte molecules with a low Raman scattering cross-section. In addition, as the SLIPS does not facilitate the adhesion of proteins in a solution, the protein biofouling study is carried onto the SLIPS and plasmonic SLIPS and compared with the plain PDMS substrate.

**Figure 1:**
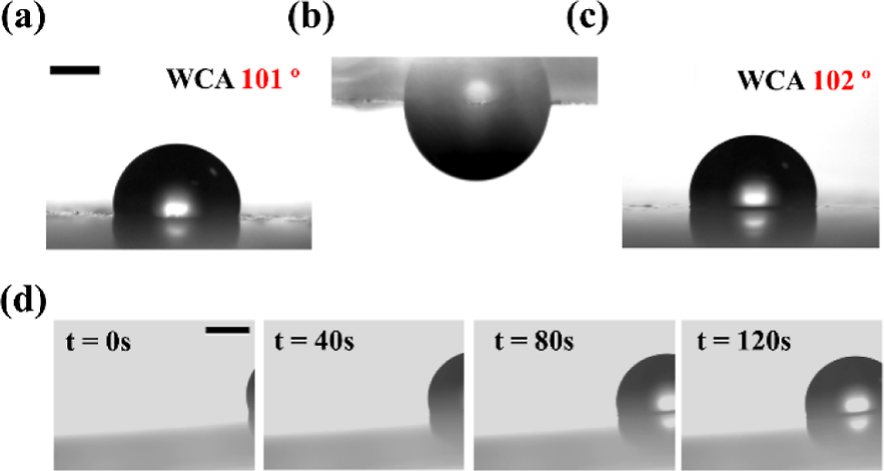
WCA of (a) PDMS, (b) slippery PDMS, and (c) water droplet pinned to the PDMS surface at 90° tilt. Scale bar: 1 mm. (d) Water droplet sliding on slippery PDMS at 5° tilt. Scale bar: 1 mm.

The commonly used PDMS substrate is fabricated normally from a two-component mixture from Dow Corning (Sylgard 184) via thermal curing of a prepolymer and a curing agent in the 10:1 ratio. During thermal curing, the Si–H group of the curing agent cross-links the monomer dimethylsiloxane through a reaction with vinyl groups (Si–CH=CH_2_) to form a 3-D polymer network.^29^ The formed solid PDMS surface is hydrophobic in nature [water contact angle (WCA) of 101 ± 3° (Figure 1a)] and exhibits good adhesion to a water droplet, as manifested in a CAH of 33° (even upon reversing the substrate, the water droplet adheres to the substrate; Figure 1b). However, thermal curing following the equal volume mixing of the silicone oil of viscosity 500 cSt with two components of the Sylgard 184 results in a nonadhesive substrate (Figure 1c, WCA at 102 ± 2°) PDMS surface but with a very low CAH of ∼1° so that a water droplet dispensed onto the surface moves with a slight tilt. The reduction in CAH can be attributed to the incorporation of silicone oil into the PDMS polymer network, which does not participate in the polymerization process. However, the presence of silicone oil makes the surface smooth and offers low adherence to the aqueous droplet. This is manifested in Supporting Information Video S1 wherein the water droplet adheres completely to the substrate even for a tilt angle of 180° and Supporting Information Video S2 wherein a tilt angle of 5° to the substrate of PDMS mixed with silicone oil results in easy movement of the water droplet (Figure 1d).

**Figure 2:**
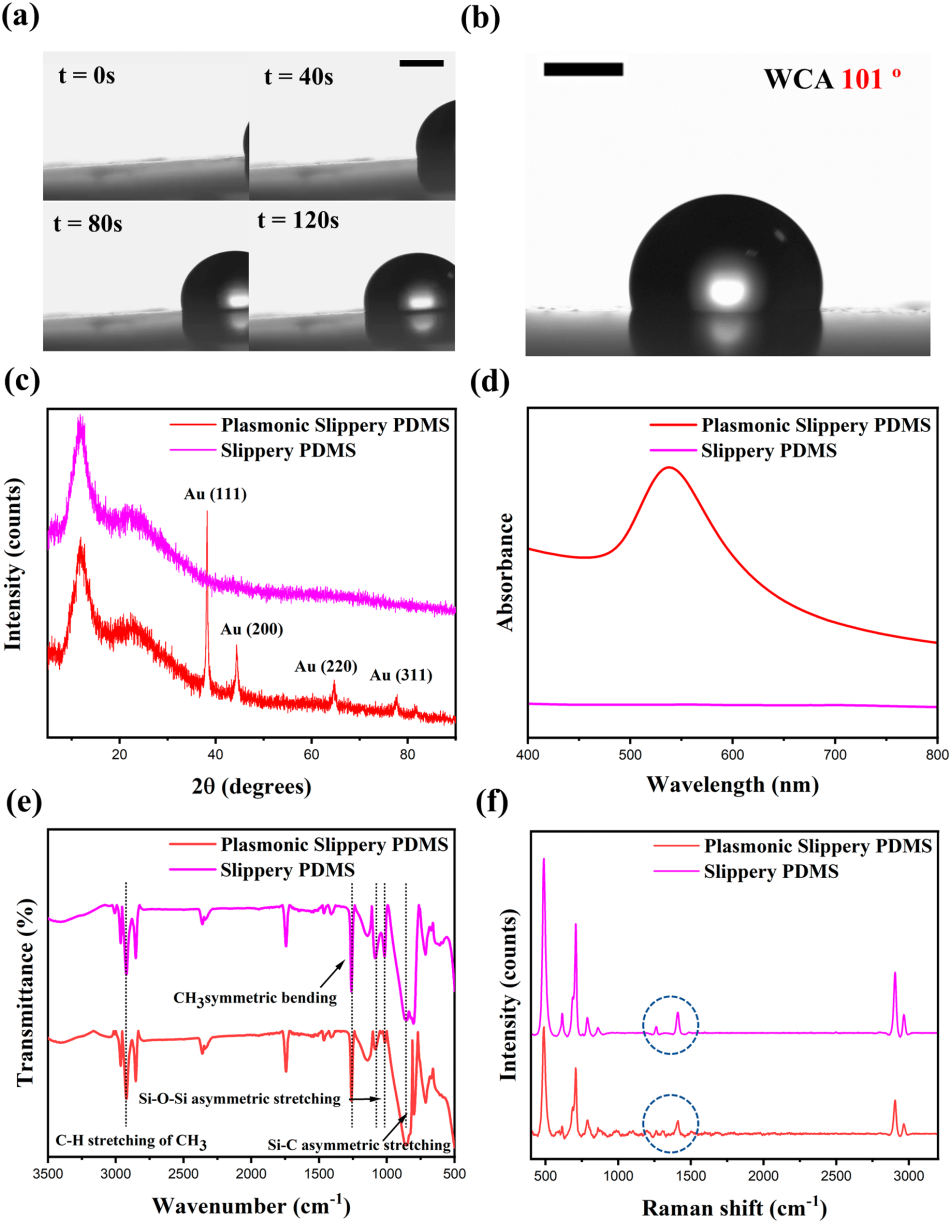
(a) 5 μL water droplet sliding at 10° tilt on plasmonic slippery PDMS. Scale bar: 1 mm. (b) WCA on plasmonic slippery PDMS at 101°. Scale bar: 1 mm. (c) XRD plot of slippery PDMS and plasmonic slippery PDMS. (d) Absorbance spectra of plasmonic slippery PDMS. (e) ATR–FTIR spectra of slippery PDMS and plasmonic slippery PDMS. (f) Raman spectra of slippery PDMS and plasmonic slippery PDMS.

The in situ reduction of the gold nanoparticles on the slippery PDMS surface was performed to create plasmonic slippery PDMS but with a change in the tilt angle from 5° to 10° (Supporting Information Video S3 and Figure 2a) while keeping its WCA ∼ 101° (Figure 2b). The slight increase in the tilt angle can be attributed to the presence of nanoparticles on the slippery surface. The in situ reduction of gold chloride to gold particles is achieved via the Si–H group’s presence in the PDMS mixture.^30^ However, the presence of a silicone oil layer at the top of the slippery PDMS makes the in situ reduction process very slow as it reduces the availability of the Si–H groups on the surface. To mitigate it, the gold chloride solution is diluted with acetone (5:1 gold chloride solution to acetone ratio) so that the surface tension of the liquid is reduced which in turn would infiltrate into the bulk PDMS matrix resulting in faster reduction of plasmonic nanoparticles. The presence of nanoparticles on the surface is confirmed via XRD studies. The XRD studies of the slippery PDMS substrate exhibit a broad peak around 12°, reflecting the amorphous nature of the substrate and the *d*-spacing of the tetragonal crystal lattice (101) of the cured PDMS.^31^ On the other hand, the plasmonic gold-reduced slippery surface exhibits additional peaks (Figure 2c) at 38.26°, 44.36°, 64.7°, 77.82°, and 81.44° corresponding to Au (111), Au (200), Au (220), Au (311), and Au (222), respectively, confirming the presence of gold on the slippery PDMS after gold reduction on the surface.^32^ In addition, the UV–vis absorption spectral measurements show the plasmonic peaks around 530–560 nm confirming the presence of gold nanoparticles on the substrate (Figure 2d).^33^ The FTIR spectroscopic studies carried out using the commercial spectrometer on the slippery and plasmonic slippery surface exhibit similar spectral features with an absorption peak at 2921 cm^–1^ corresponding to C–H stretching in the methyl group; 1261 cm^–1^ corresponding to CH_3_ symmetric bending; and 1086 and 1016 cm^–1^ corresponding to Si–O–Si asymmetric stretching.^34^ It is pertinent to note that the intensity of the Si–O–Si asymmetric stretching at 1086 and 1016 cm^–1^ is diminished in plasmonic slippery PDMS (Figure 2e). To investigate if any chemical changes occur to silicone oil heated at 80 °C and to silicone oil after mixing with acetone, 1 mL of silicone oil and 1 mL of acetone were magnetically stirred for 15 min and the mixture was left for 24 h for solvent evaporation before spectra of silicone oil were recorded. FTIR analysis revealed characteristic silicone oil peaks: 1150–1000 cm^–1^ peaks corresponding to the Si–O–Si bond; 1260 cm^–1^ peaks corresponding to the CH_3_ symmetric deformation of the Si–CH_3_ bond; 1410 cm^–1^ peaks corresponding to the CH_3_ asymmetric deformation of the Si–CH_3_ bond; and 2963 cm^–1^ peaks corresponding to the C–H stretching of the CH_3_ bond in silicone oil. It is observed that these peaks are similar for the silicone oil heated at 80 °C for 24 h as well as for the silicone oil mixed with acetone (Supporting Information Figure S1). No additional peaks were observed with heating or addition of acetone, indicating no chemical changes in silicone oil compared to untreated silicone oil. Further, the Raman spectral measurements are carried out using a micro-Raman spectrometer setup explained in our previous work.^35^ The Raman spectra of the slippery PDMS and plasmonic slippery PDMS are observed to be similar except with diminished peaks at 1261 and 1411 cm^–1^ for plasmonic slippery PDMS, corresponding to methyl symmetric and asymmetric bending (Figure 2f).

**Figure 3:**
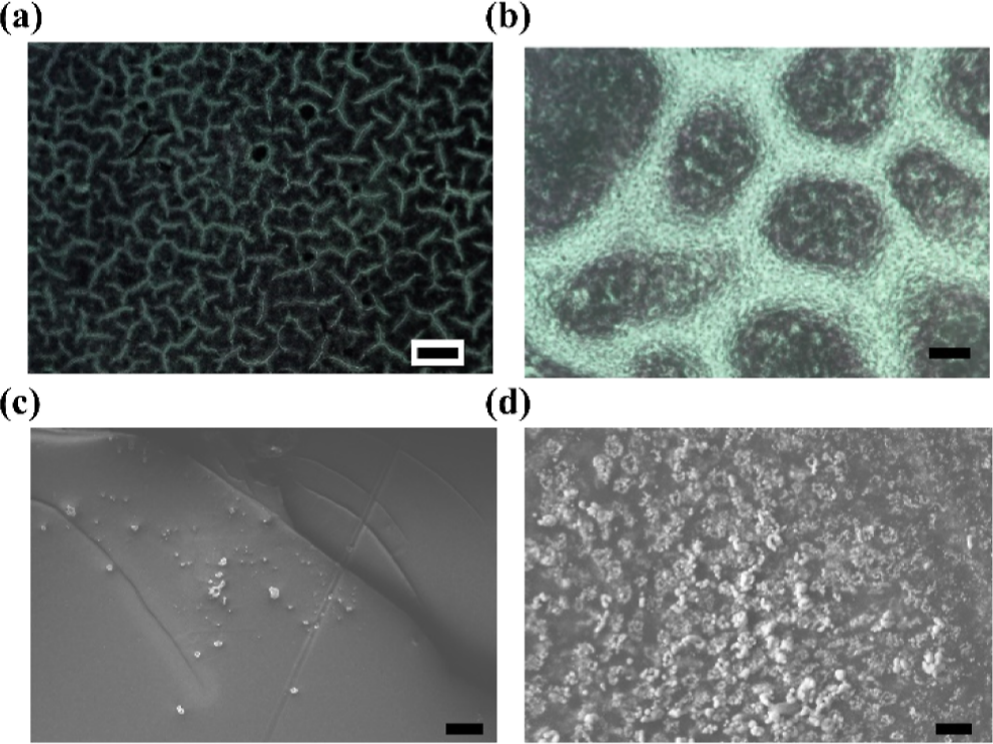
(a) Optical image of plasmonic slippery PDMS at scale 100 μm. (b) Optical image of plasmonic slippery PDMS at scale 10 μm. (c) SEM image of slippery PDMS. Scale: 2 μm. (d) SEM image of plasmonic slippery PDMS. Scale: 2 μm.

The optical images of the plasmonic slippery PDMS suggest wrinkle-like patterns containing gold nanostructures (Figure 3a). As observed in the optical microscopic image of the plasmonic slippery PDMS surface (Figure 3b), the substrate comprises of gold nanoparticle-/nanostructure-reduced region and silicone oil region. The silicone oil region where the nanoparticles/nanostructures are not reduced contributes to the slippery behavior, whereas the gold-reduced PDMS region act as a potential plasmonic-active region. Further, SEM images of slippery PDMS and plasmonic slippery PDMS are shown in Figure 3c,d. From the figure, it can be observed that 48 h of gold reduction led to gold nanoparticle-/nanostructures on the slippery PDMS. The prospects of the fabricated surface as the SERS substrate are supported from the studies by Schellenberger et al.,^36^ where it was observed that a droplet resting on a liquid-infused surface comes in contact with underlying surface structures (in this case, gold nanostructures) even though the lubricant liquid layer was present on such surfaces.

**Figure 4:**
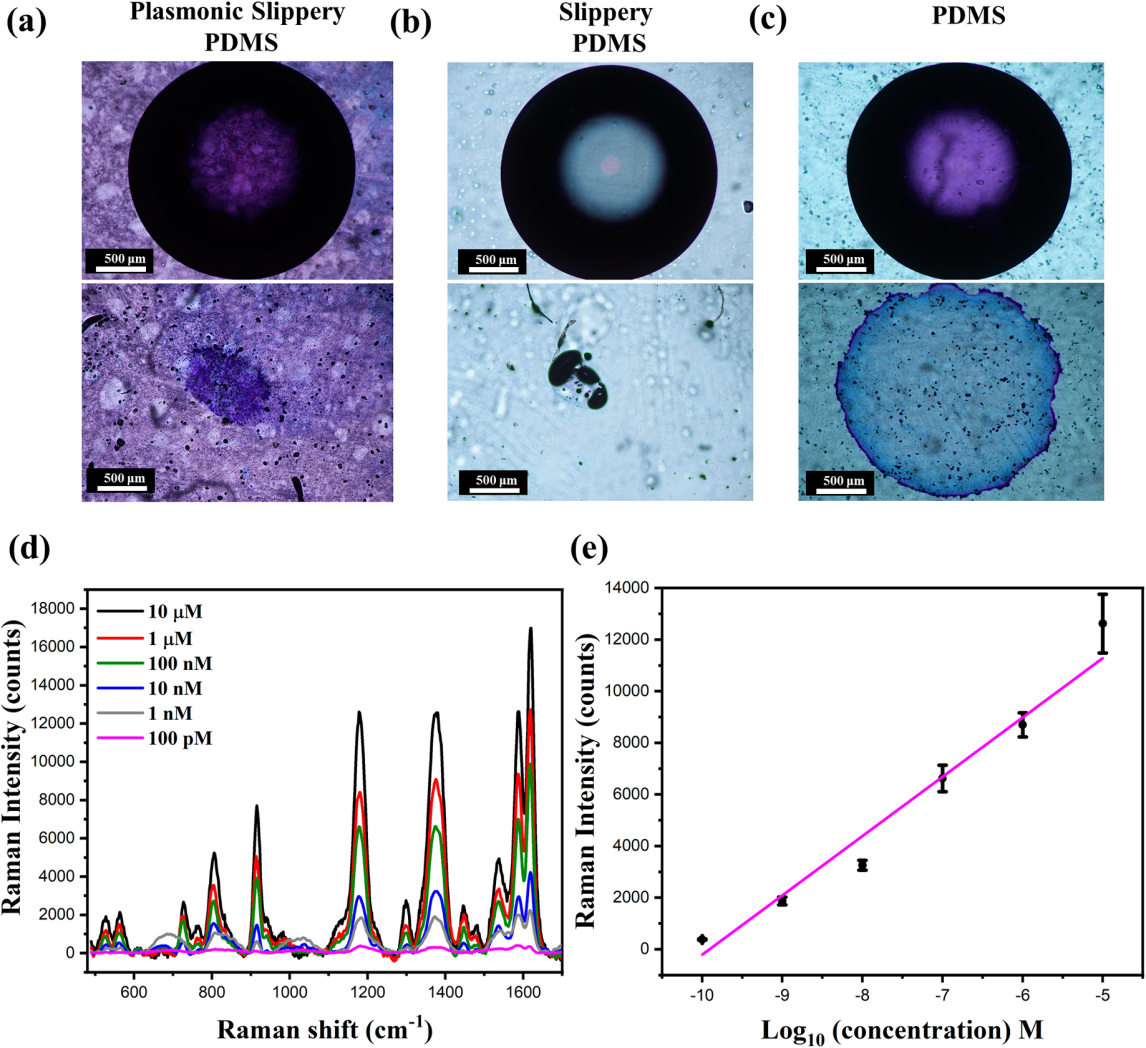
Pinning-free evaporation of crystal violet solution on (a) plasmonic slippery PDMS, (b) slippery PDMS, and (c) coffee-ring stain of the crystal violet solution on PDMS. Scale bar: 500 μm. (d) Backscattered Raman spectra of crystal violet of varied concentration. (e) Concentration vs Intensity plot for the peak at 1376 cm^–1^ Raman shift.

The potential of the plasmonic slippery surface for SERS detection is investigated by drying a 5 μL droplet of crystal violet onto the substrate. Due to low CAH, the evaporation of the crystal violet is pinning free as no coffee-ring effect is observed here (Figure 4a) as in the case of slippery PDMS (Figure 4b). But the high CAH surface of PDMS resulted in coffee-ring stain (Figure 4c). Herein, a 532 nm diode laser (power ∼6 mW) is focused through a 4× microscope objective to excite the dried crystal violet and the backscattered Raman signal is collected through the same objective and coupled to the spectrograph–CCD assembly (Horiba Scientific, IHR320-Symphony II CCD assembly, slit width 0.1 mm, and a spectral acquisition time of 20 s). The crystal violet-concentration-dependent Raman signal obtained is shown in Figure 4d. The obtained Raman spectral peaks corresponding to crystal violet appear at 523 cm^–1^, 563 cm^–1^, 724 cm^–1^, 759 cm^–1^, 805 cm^–1^, 915 cm^–1^, 1178 cm^–1^, 1297 cm^–1^, 1376 cm^–1^, 1445 cm^–1^, 1478 cm^–1^, 1535 cm^–1^, 1587 cm^–1^, and 1617 cm^–1^, respectively, agreeing with the literature.^37,38^ The LOD is estimated by the following equations, LOD = antilog_10_(*x*), where , where *y*_0_ and σ are the intensity count at zero analyte concentration and corresponding standard deviation (SD), respectively, *c* is the *y*-intercept, and *m* is the slope of the linear fit. The LOD for crystal violet is obtained by fitting the 1376 cm^–1^ Raman peak intensity as a function of concentration and is found to be 76 pM (Figure 4e). The plasmonic effect of the substrate is further substantiated by the fluorescence quenching of crystal violet by the substrate (Supporting Information Figure S2) via well-known nonradiative energy transfer to the plasmonic surfaces. Further, the Raman studies on plasmonic slippery PDMS and slippery PDMS are compared with high-concentration CV solutions and low-concentration CV solutions. From Supporting Information Figure S3, it can be observed that on plasmonic slippery PDMS, the Raman signals from high-concentration CV solution (3 mM, 2 mM, and 1 mM) exhibited comparable Raman intensities indicating bulk contribution from the CV residue, whereas low-concentration CV solutions (100 μM and 10 μM) exhibited Raman signal intensities higher than the Raman signals from high-concentration CV solutions, indicating SERS effect from the plasmonic slippery PDMS for concentrations less than 100 μM crystal violet solutions. In contrast, the Raman signals from low-concentration CV solutions were significantly of lower intensities on slippery PDMS. The enhancement factor (EF) of the approach is measured by comparing the Raman signal of the dye concentrations on a plain slippery PDMS substrate without gold reduction. The equation for calculating the EF is given by
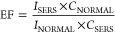
where *I*_SERS_ and *I*_NORMAL_ are the peak intensities of the characteristic band from SERS and normal Raman measurements, respectively, and *C*_NORMAL_ and *C*_SERS_ are the concentrations of analyte used in the normal Raman and SERS measurements, respectively. It is found that the method has achieved an EF of 10^4^ for crystal violet. Further, the SERS activity of the plasmonic slippery PDMS is compared with a nonslippery plasmonic PDMS. The nonslippery plasmonic PDMS is prepared by immersing the PDMS in 20 mM gold chloride solution for 48 h. The nonslippery PDMS exhibited a large coffee-ring residue as shown in Supporting Information Figure S4 and the crystal violet sensing was limited to 10 μM concentration as shown in Supporting Information Figures S5 and S6. The Raman signal intensity counts for plasmonic slippery PDMS, slippery PDMS, and nonslippery plasmonic PDMS for 10 μM CV solution are 12462 ± 374, 4206 ± 797, and 26 ± 4 counts, respectively, at peak 1376 cm^–1^ (Supporting Information Figure S7), indicating the significance of concentration enrichment of the plasmonic slippery surface.

**Figure 5:**
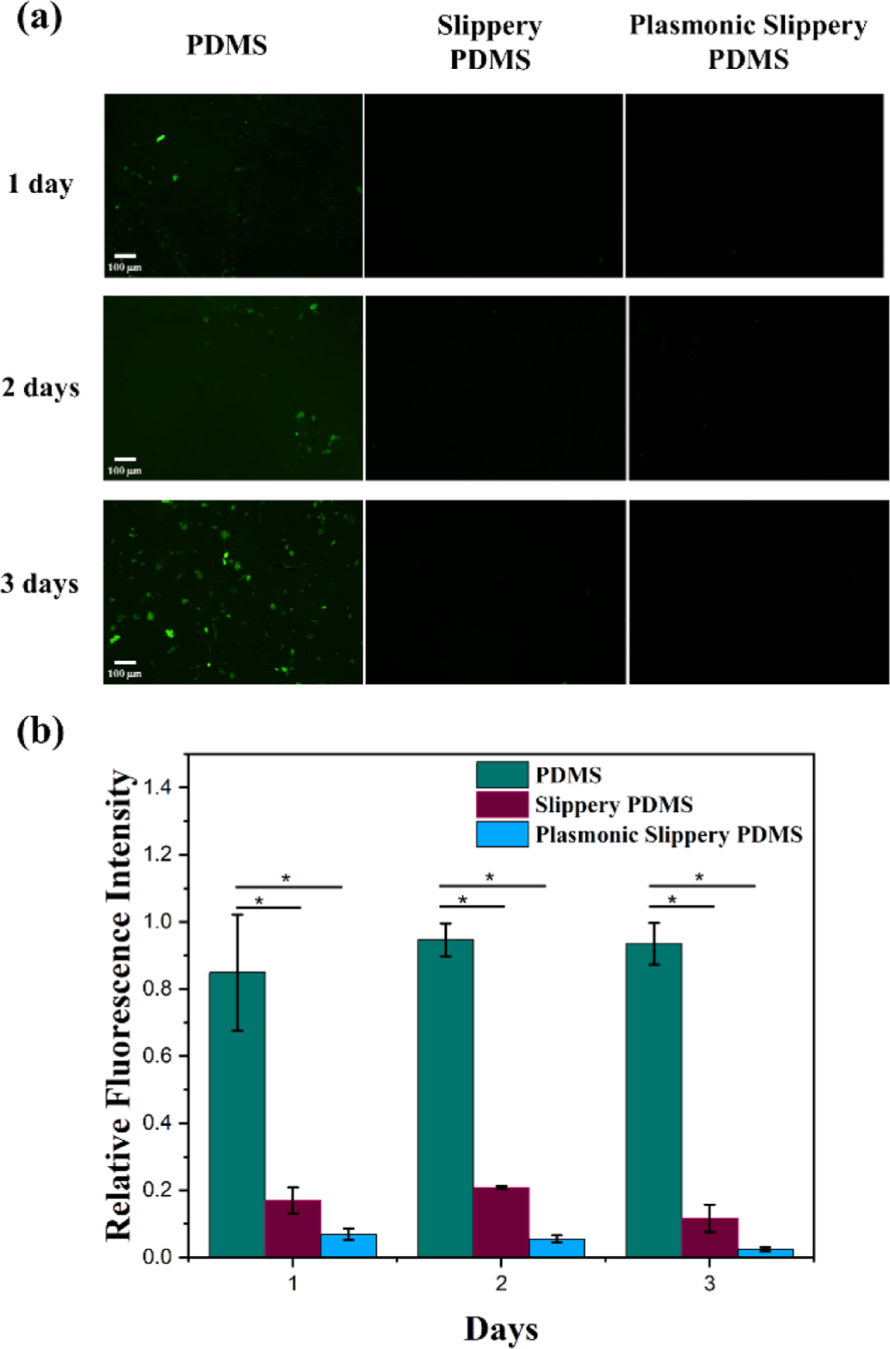
(a) Fluorescence microscopy images of FITC-BSA adhesion on PDMS, slippery PDMS, and plasmonic slippery PDMS for 1 day, 2 days, and 3 days. Scale bar: 100 μm. (b) Relative fluorescence intensity on the substrate. Error bars represent the mean ± SD. An * indicates statistical significance compared to the control groups at the level of *p* < 0.05 using ANOVA followed by a posthoc test. *N* = 3.

The understanding of protein–surface interaction is fundamental to addressing many questions in biology and medicine such as the tendency of the blood to coagulate, the efficacy of the implant materials after surgery, etc. Apart from electrostatic and van der Waals forces, the hydrophobic interactions are also reported to play an important role in the adsorption of the protein to the surface. It is believed that the hydrophobic surfaces lead to more denaturation of the protein than hydrophilic surfaces. Furthermore, dehydration of a surface must occur for proteins to adsorb. On hydrophobic substrates, this results in a reduction of Gibb’s free energy and increases protein adsorption. In the case of adsorption on hydrophilic and charged materials, electrostatic and van der Waals interactions are the primary cause of protein binding to surfaces.^39,40^ Herein, as the substrate is hydrophobic, the hydrophobic forces are the major driving force of protein adsorption in PDMS. To investigate the protein adsorption onto the prepared samples, herein, we investigated the adsorption of a model protein BSA on slippery PDMS and plasmonic slippery PDMS surfaces using fluorescence microscope spectroscopy. Herein, the fabricated substrates are immersed inside a beaker containing FITC-BSA (1 mg/mL) in PBS solution for 1 day, 2 days, and 3 days (Figure 5a). In general, complex protein adsorption involves various kinds of protein–substrate interactions and is influenced by different factors that depend upon the surface properties and environment. As the days of incubation of the substrate in protein solution increases, the strength of the fluorescence signal increases, indicating the more efficient adsorption of proteins with time on PDMS. On the other hand, the fluorescence signals are diminished in slippery PDMS and plasmonic slippery PDMS. Herein, the protein used BSA as a globular protein with 583 amino acid residues with 17 intrachain disulfide bonds and one free thiol group. Its secondary structure consists of approximately 54% α-helix and 40% β-structure (β-sheet plus β-turns) and contains three binding domains, which are specific for metal ions, lipids, and nucleotides, respectively. On hydrophobic surfaces such as PDMS, protein absorption is driven by the attraction of the nonpolar parts of BSA molecules toward the surface. A monolayer of BSA is reported to occur within a time scale of less than 2 h for concentrations less than 10 mg/mL. With the increase in incubation time, crowding of BSA molecules within the monolayer occurs due to changes in the inherent flexibility of the protein molecule. However, the relative fluorescence intensity analysis indicated that compared with PDMS, slippery PDMS and plasmonic slippery PDMS showed significantly reduced fluorescence (Figure 5b). In addition, SERS analysis was conducted on plasmonic slippery PDMS and nonslippery plasmonic PDMS after 24 h incubation in BSA–PBS solution (10 mg/mL). As shown in Supporting Information Figure S8, no additional Raman signal from BSA was observed from the surface, indicating protein absorption inhibition on the surface, whereas protein fouling was evident in plasmonic PDMS. These results suggest that the prepared slippery PDMS and plasmonic slippery PDMS have excellent antibiofouling properties against proteins even after the reduction of gold nanostructures onto the surface.

## Conclusions

In summary, herein, we illustrated a simple and facile fabrication of surfaces similar to SLIPSs that exhibit ultralow CAH and tilt angle and were converted into a plasmonic substrate via in situ reduction of gold nanoparticles. The potential of such plasmonic SLIPSs without a coffee-ring effect as a sensitive analytical platform was demonstrated by illustrating the quantitative detection of crystal violet down to ∼76 pM. Further, the protein adsorption studies demonstrated that the plasmonic SLIPS inhibits the model protein, BSA, adsorption to a large extent, and the efficacy of inhibition was comparable with that of slippery PDMS. With all of the capabilities of the fabricated plasmonic surface, we anticipate that it could be developed to meet the emerging needs in ultrasensitive biofluid detection for clinical applications, environmental pollution monitoring, food safety application, and to create antiprotein fouling surfaces.

## Supporting Information Available

5 μL water droplet pinning on PDMS at 180° tilt (MP4)
5 μL water droplet sliding at 5° tilt on slippery PDMS (MP4)
5 μL water droplet sliding at 10° tilt on plasmonic slippery PDMS
(MP4)
FTIR spectra of silicone oil, silicone oil heated at 80 °C, and silicone oil mixed with acetone; fluorescence quenching on plasmonic slippery PDMS coating using crystal violet as an analyte through spectroscopic analysis; SERS effect of plasmonic slippery PDMS on various crystal violet concentrations compared to slippery PDMS; study conducted with 785 nm laser with acquisition time 2 s; evaporation residue on plasmonic slippery PDMS and nonslippery plasmonic PDMS; SERS detection limit on nonslippery plasmonic PDMS and slippery PDMS; SERS detection limit on nonslippery plasmonic PDMS and slippery PDMS; Raman spectra of various crystal violet concentrations on nonslippery plasmonic PDMS, nonslippery plasmonic PDMS (for lower concentrations), slippery PDMS, and slippery PDMS (for lower concentrations); standard deviation of Raman signal intensity from 10 μM crystal violet residue on nonslippery plasmonic PDMS, slippery PDMS, and plasmonic slippery PDMS; and Raman signals from nonslippery plasmonic PDMS and plasmonic slippery PDMS after 24 h incubation in BSA–PBS solution (10 mg/mL) (PDF)
