# Supplementary material for: Plasmonic Slippery Surface for Surface-Enhanced Raman Spectroscopy and Protein Adsorption Inhibition
Source: Anal Chem. 2025 Jan 28;97(5):2610–7. doi: 10.1021/acs.analchem.4c01844 (PMC11822738; doi:10.1021/acs.analchem.4c01844)
Supplement: Supplementary file 4 — ac4c01844_si_004.pdf [file ac4c01844_si_004.pdf]

Supporting Information

Plasmonic slippery surface for surface-enhanced Raman spectroscopy and protein adsorption inhibition

Swithin Hanosh[a], Monisha K[a], Sajan D. George[a,b*]

[a] Department of Atomic and Molecular Physics, Manipal Academy of Higher Education, Manipal 576104, India.
[b] Centre for Applied Nanosciences (CANs), Manipal Academy of Higher Education, Manipal 576104, India.
* *Corresponding Author E-mail:* sajan.george@manipal.edu

Contents

S1. FTIR spectra of silicone oil, silicone oil heated at 80 ºC, and silicone oil mixed with acetone…………………………………………………….……….…….……….………..….…**S2**

S2. Fluorescence quenching on plasmonic slippery PDMS……….……….……….……..….…**S3**

S3. SERS effect of plasmonic slippery PDMS on various crystal violet concentrations compared to slippery PDMS. Study conducted with 785 nm laser with acquisition time 2s……….……….……….…………………………………………………………………………**S3**

S4. Evaporation residue on plasmonic slippery PDMS and non-slippery plasmonic PDMS……………………………………………………………………....…………..…..…**S4**

S5. SERS detection limit on non-slippery plasmonic PDMS and slippery PDMS………………………………………………………………………….……….…..…**S4**

S6. SERS detection limit on non-slippery plasmonic PDMS and slippery PDMS. Raman spectra of various crystal violet concentrations on a) non-slippery plasmonic PDMS, b) non-slippery plasmonic PDMS (for lower concentrations), c) slippery PDMS d) slippery PDMS (for lower concentrations) ………………………………………………………………………..…**S5**

S7. Standard deviation of Raman signal intensity from 10 μM crystal violet residue on non-slippery plasmonic PDMS, slippery PDMS, and plasmonic slippery PDMS……………………………..**S6**

S8. Raman signals from plasmonic slippery PDMS after 24 hours immersion in BSA-PBS solution (10 mg/ml) ……………………………………..…………….………….…..……..…**S6**

S9. Movie legends……………………………………………………………...……….……..**S7**

S1. FTIR spectra of silicone oil, silicone oil heated at 80 °C, and silicone oil mixed with acetone.

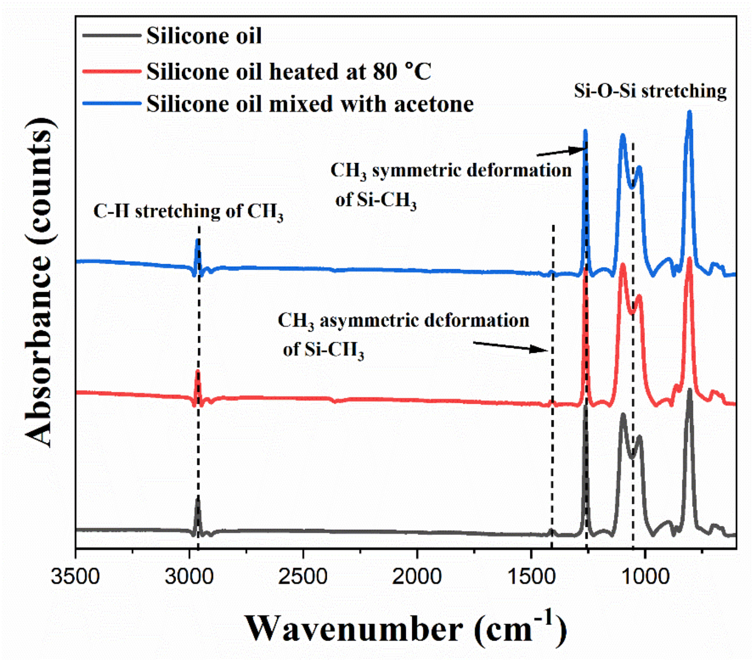

S2. Fluorescence quenching on plasmonic slippery PDMS coating using crystal violet as analyte through spectroscopic analysis.

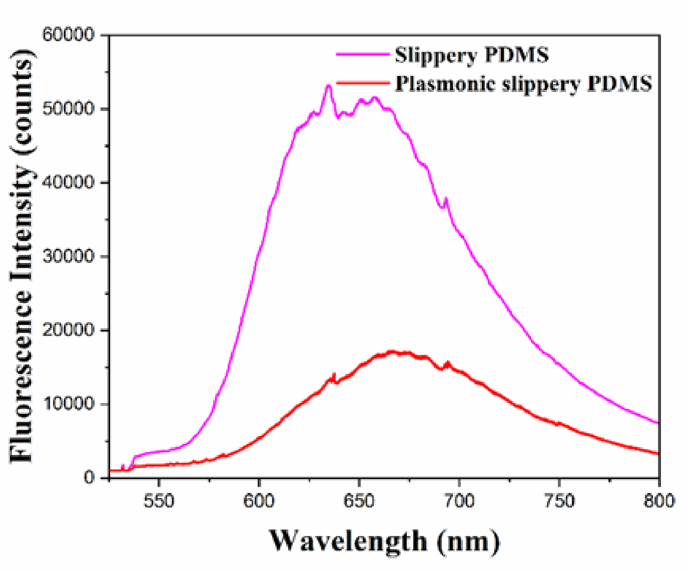

S3. SERS effect of plasmonic slippery PDMS on various crystal violet concentrations compared to slippery PDMS. The Raman study was conducted with 785 nm laser with an acquisition time of 2s.

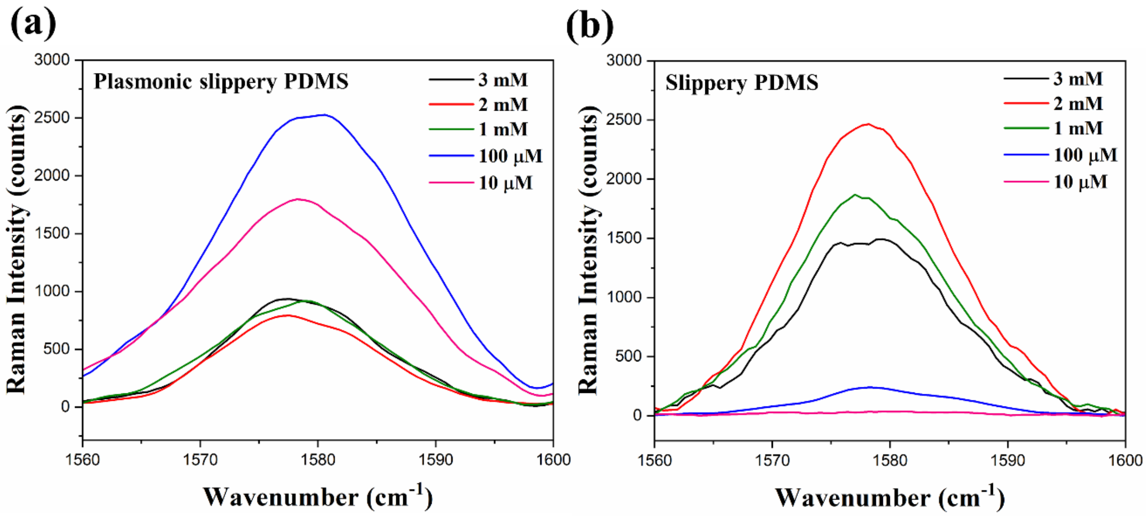

S4. Evaporation residue on (a) plasmonic slippery PDMS (b) non-slippery plasmonic PDMS.

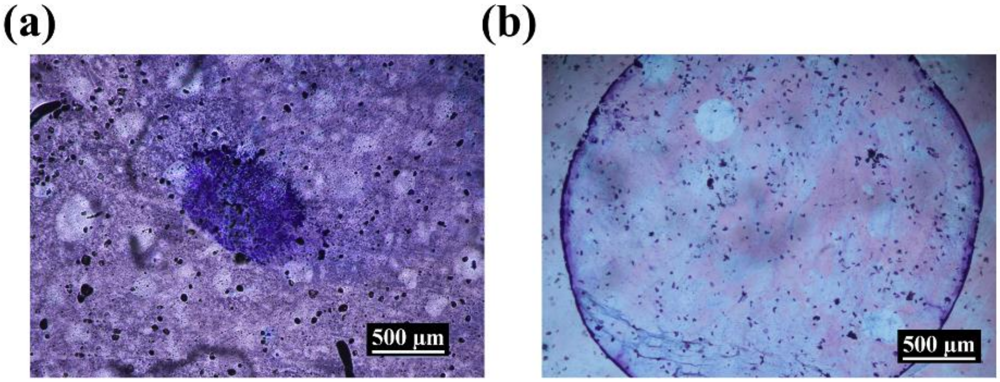

S5. SERS detection limit on non-slippery plasmonic PDMS and slippery PDMS.

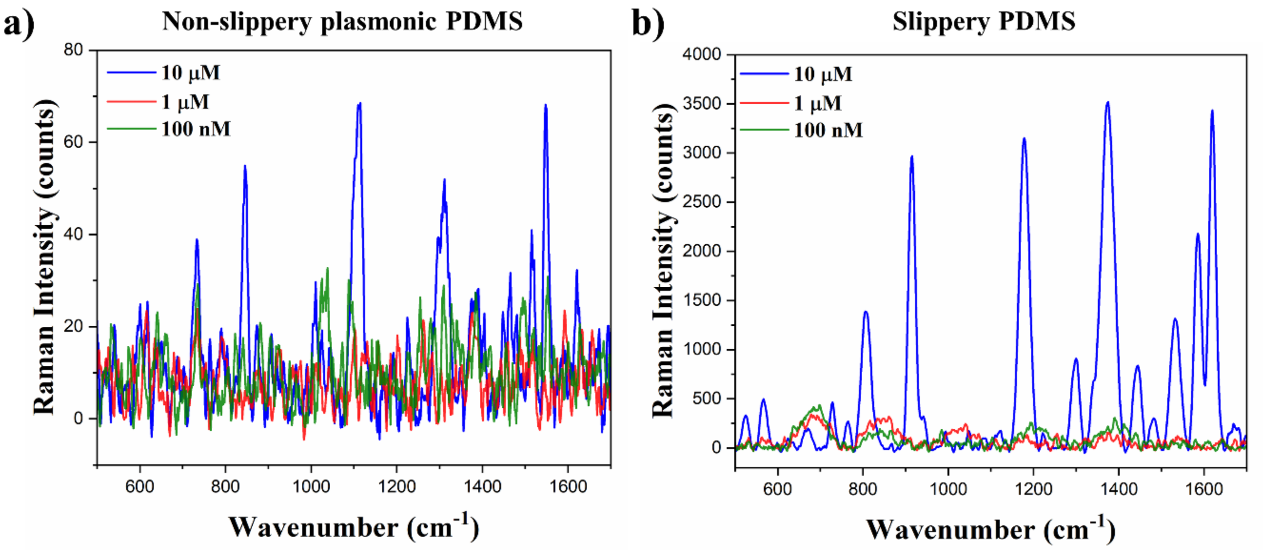

S6. SERS detection limit on non-slippery plasmonic PDMS and slippery PDMS. Raman spectra of various crystal violet concentrations on a) non-slippery plasmonic PDMS, b) non-slippery plasmonic PDMS (for lower concentrations), c) slippery PDMS d) slippery PDMS (for lower concentrations). Raman studies conducted with 785 nm laser for an acquisition time of 20s on the non-slippery plasmonic PDMS. Due to Raman signals saturating from CV solution on slippery PDMS, the studies couldn't be realized with the same acquisition time. Hence, Raman signal of CV from slippery PDMS were conducted at an acquisition time of 2s.

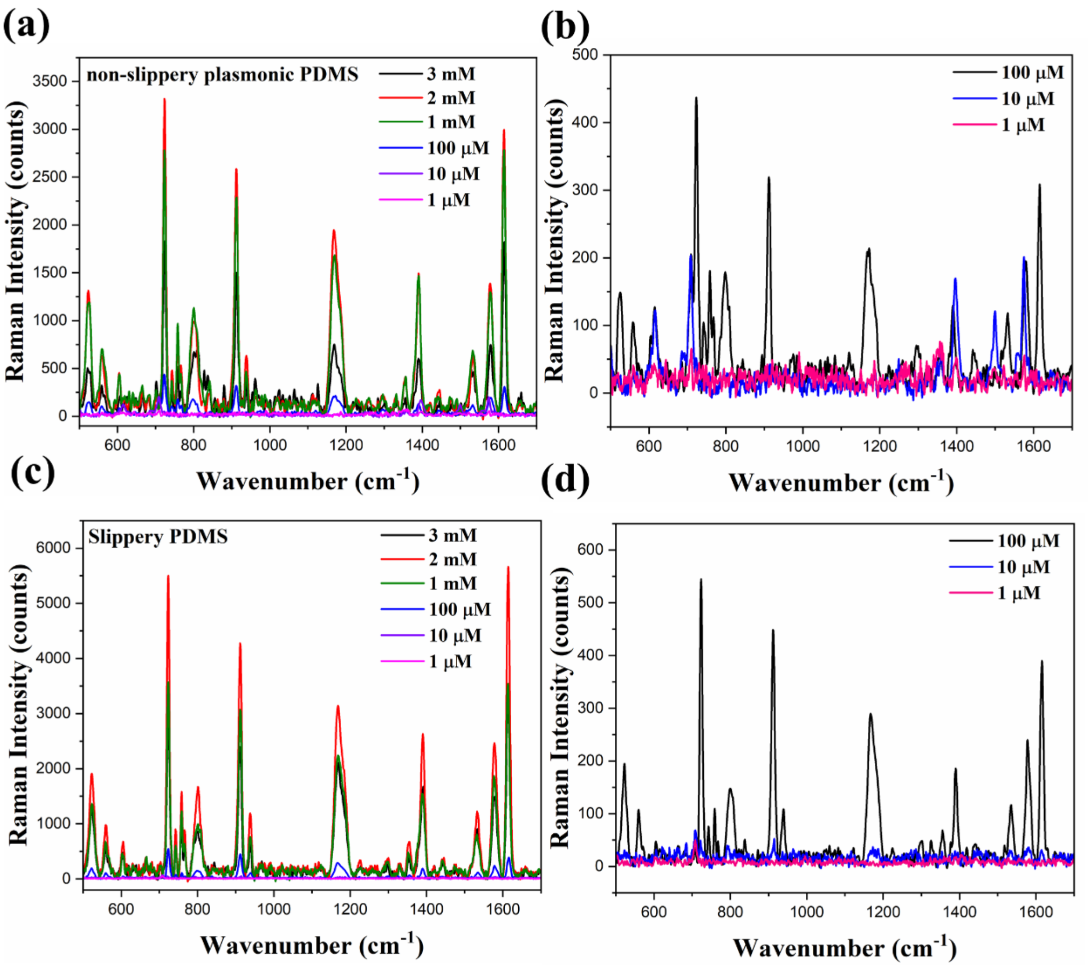

S7. Standard deviation of Raman signal intensity from 10 µM crystal violet residue on non-slippery plasmonic PDMS, slippery PDMS, and plasmonic slippery PDMS.

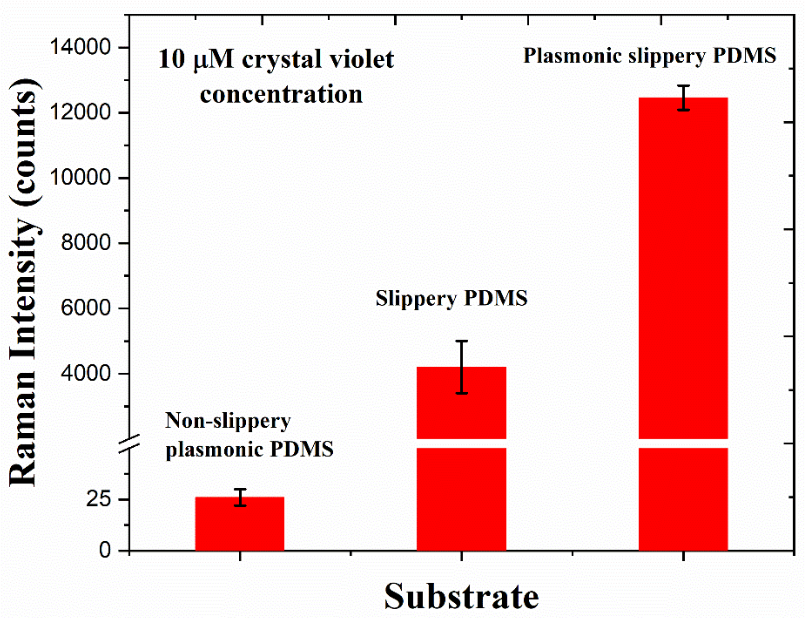

S8. Raman signals from non-slippery plasmonic PDMS and plasmonic slippery PDMS after 24 hours incubation in BSA-PBS solution (10 mg/ml). Peak at 1090 cm^{-1} corresponding to stretching vibration of the C-N bond in the phenylalanine residues from BSA were observed on the surface indicating protein fouling on non-slippery plasmonic PDMS.

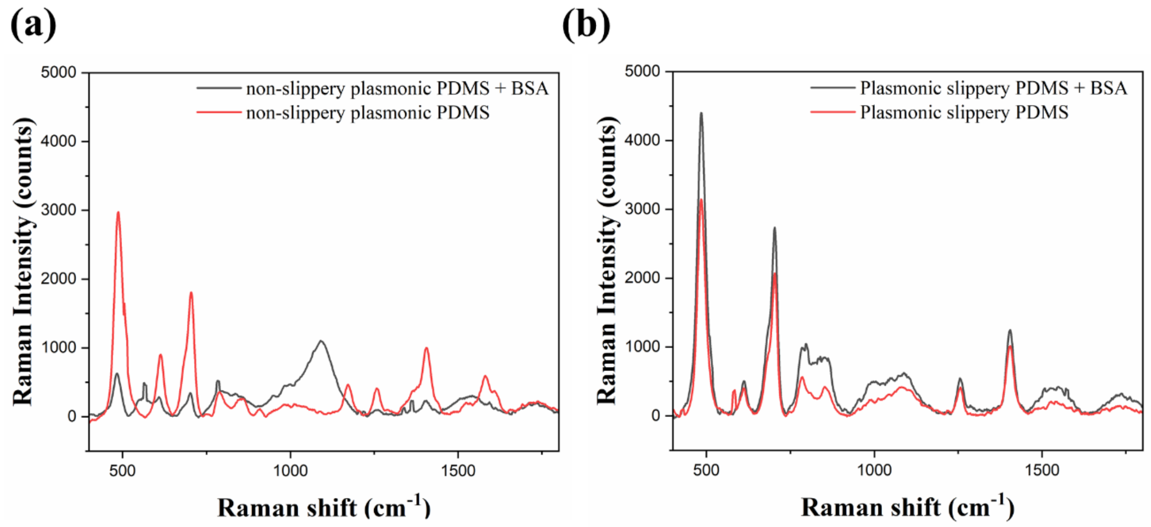

S9. Movie legends

Supplementary Video S1: 5 µl water droplet pinning on PDMS at 180 º tilt.

Supplementary Video S2: 5 µl water droplet sliding at 5 º tilt on slippery PDMS.

Supplementary Video S3: 5 µl water droplet sliding at 10 º tilt on plasmonic slippery PDMS.
